# Computational Quantification of Collagen Density and Ki67‐Positive Cells in a Forensic Porcine Wound Model

**DOI:** 10.1111/apm.70217

**Published:** 2026-05-08

**Authors:** Kristiane Barington, Christof Albert Bertram, Katharina Breininger, Henrik Elvang Jensen

**Affiliations:** ^1^ Department of Veterinary and Animal Sciences, Faculty of Health and Medical Sciences University of Copenhagen Frederiksberg C Denmark; ^2^ Centre of Pathobiology University of Veterinary Medicine Vienna Vienna Austria; ^3^ Center for AI and Data Science Julius‐Maximilians‐Universität Würzburg Würzburg Germany

**Keywords:** age assessment, computational pathology, porcine, veterinary forensic pathology, wound

## Abstract

Obtaining an accurate age of skin wounds is a diagnostic challenge in forensic pathology. This study aimed to quantify collagen density and proliferation activity in porcine experimental wounds over time using machine learning‐based segmentation to provide an objective method for wound age estimation. Tissue sections from porcine experimental wounds (*n* = 68) were stained with Masson's trichrome stain or immunohistochemically labeled for proliferation activity by Ki67. The experimental wounds were located on the back and 5–35 days old. Collagen and proliferation activity in the wounds were quantified by training and application of neural network pixel and random trees object classifiers. The relative collagen fraction and the collagen ratio between lower and upper wound regions displayed significant time‐dependent patterns. The proliferative activity, assessed by the percentage of Ki67‐positive cells, was not suitable for age assessment. In conclusion, the application of a neural network pixel classifier trained to differentiate between collagen and cellular components is an objective method for forensic wound age assessment. However, to obtain a higher precision, the method should be used as a supportive tool in combination with other time‐dependent markers.

## 1 Introduction

Neglect of animals with severe wounds is considered a violation of European legislation on the protection of animals [1]. In such cases, the wound age estimated by a veterinary pathologist may be used to evaluate the extent of neglect and may result in legal consequences for the accused [2, 3, 4]. Therefore, age assessment should hold the highest degree of accuracy and precision and be based on objective methods [5]. However, due to the limited number of scientific studies of wound healing from a forensic perspective, wound age is assessed in broad time intervals [3]. In Denmark, the majority of lesions are typically estimated to be several weeks old, but the lack of precision makes it difficult to assess the degree of neglect [3].

Currently, wound age assessment in pigs is based on granulation tissue thickness and a histological assessment [6]. However, histological evaluation and interpretation are affected by intra‐ and interobserver variability [5, 6, 7]. Moreover, the thickness of granulation tissue cannot be used for age assessments as the only parameter, as shown in an experimental porcine wound model [5, 8]. Therefore, the development of objective methods assessing wound age in veterinary forensic pathology is warranted. Computational pathology has proven useful for segmenting porcine intestinal wall and quantifying cells in porcine bruises [9, 10]. However, few studies have evaluated how computational pathology, such as the application of cell detection algorithms, can be used for age assessments of wounds in a forensic context [5, 11].

Wound healing is typically divided into four sequential and overlapping phases: (1) hemostasis, (2) inflammation, (3) proliferation, and (4) maturation [12, 13]. The proliferative phase of wound healing is characterized by angiogenesis, fibroblast proliferation, and collagen deposition, forming granulation tissue [12, 14]. Mitotic activity of epithelial cells showed a time‐dependent development in experimental dry‐ice burn wounds in rats [15]. However, it has not yet been investigated whether proliferation activity in granulation tissue is useful for age assessments of porcine wounds.

Collagen deposition in the wound bed has been shown to reflect wound age in pigs, dogs, guinea pigs, rats, and humans [2, 8, 16, 17, 18]. In canine and porcine wounds stained with Van Gieson stain and Masson's trichrome stain (MT), collagen fibers were shown to develop from thin fibers in the youngest wounds to thicker bundles as wound age increased [2, 8]. However, the evaluation of the new collagen was either descriptive or evaluated semiquantitatively [2, 8]. None of the studies accounted for interobserver variability and variations in slide thickness and staining intensity. To implement collagen evaluation as a tool for wound age assessment in forensic cases, an objective method is needed.

This study aimed to quantify the amount of collagen and proliferation activity in porcine experimental wounds over time using machine learning‐based segmentation, including training and application of artificial neural network pixel and random tree object classifiers. Moreover, the ratio between the amount of collagen formed in the lower and upper parts of the wounds was evaluated for its application in wound age estimation.

## 2 Materials and Methods

### 2.1 Tissue Sections

Hematoxylin and eosin (HE) stained tissue sections of experimental wounds (*n* = 68) from 24 pigs were included in the present study. The experimental porcine wound model was approved by the Danish Animal Inspectorate (2023‐15‐0201‐01363). In brief, experimental pigs with a body weight of 20–25 kg were anesthetized, and four full‐thickness wounds were excised on the back and left to heal by second intention for 5, 10, 15, 20, 25, 30, and 35 days before euthanasia. After euthanasia, wound tissue was sampled from the center of the wounds, fixed in 10% buffered formalin, processed through stepwise concentrations of ethanol and xylene, before paraffin‐embedding. From each wound, a 5 μm tissue section was cut, mounted on a glass slide, and stained with HE [5].

For the present study, the HE‐stained tissue slides were destained and re‐stained as described in the subsequent sections. First, tissue slides were placed in xylene for two days, and the cover slips were removed. Thereafter, tissue sections were processed through stepwise concentrations of ethanol (99%, 96%, and 70%), destained under visual control in a 70% ethanol solution with 0.37% hydrogen chloride, and rinsed in water.

### 2.2 Ki67

Ki67 is a nuclear protein expressed in proliferating cells in the mid G_1_, S, G_2_, and M phases of the cell cycle [19]. Immunohistochemistry was performed on destained tissue sections from 20 experimental wounds (*n* = 20 pigs) (Table 1). Tissue sections were deparaffinized and rehydrated to water before immunohistochemical labelling. Porcine intestinal tissue was included as a positive control. Antigen retrieval was performed in T‐EG buffer (pH 8.0) 2 × 5 min, followed by a 15‐min equilibration in the same buffer. The sections were then rinsed in TBS (pH 7.6) for 2 × 5 min. Endogenous peroxidase activity was blocked by incubation in 0.6% hydrogen peroxide for 15 min, after which the slides were washed again in TBS for 2 × 5 min. Non‐specific binding was blocked using 2.5% normal goat serum for 5 min.

**TABLE 1:** Total number of wounds (pigs) in each of the experimental age groups. Wounds were stained with either Masson's trichrome stain (MT) to assess collagen or Ki67 immunohistochemistry to evaluate cell proliferation.

Wound age	MT	Ki67
5 days	6 wounds (3 pigs)	2 wounds (2 pigs)
10 days	8 wounds (4 pigs)	3 wounds (3 pigs)
15 days	8 wounds (4 pigs)	3 wounds (3 pigs)
20 days	8 wounds (4 pigs)	3 wounds (3 pigs)
25 days	8 wounds (4 pigs)	3 wounds (3 pigs)
30 days	4 wounds (2 pigs)	3 wounds (3 pigs)
35 days	6 wounds (3 pigs)	3 wounds (3 pigs)
Total	48 wounds (24 pigs)	20 wounds (20 pigs)

The primary antibody, Ki67 (clone MIB‐1, Dako M7240), was applied at a dilution of 1:200 in 1% BSA/TBS and incubated overnight at 4°C. For negative controls, an IgG1 isotype control (Dako X0931) diluted 1:250 in 1% BSA/TBS was used under identical conditions. Following primary incubation, the sections were rinsed in TBS for 2 × 5 min and subsequently incubated for 30 min with the ImmPRESS HRP Goat Anti‐Mouse IgG Polymer Reagent (Vector Laboratories, MP‐7452). After an additional TBS wash (2 × 5 min), color development was achieved using AEC substrate (Vector Laboratories, SK‐4200) for 10 min. Slides were then rinsed in running tap water for 2 × 5 min and counterstained with Mayer's hematoxylin for 30 s, followed by rinsing in distilled water for 2 × 5 min. Finally, the sections were mounted in warmed glycerol‐gelatin mounting medium.

TBS (pH 7.6) was prepared by dissolving 14.54 g Tris–HCl, 3.449 g Tris base, and 24.0 g NaCl in 4 L of deionized water, followed by adjustment of the pH to 7.6. The 0.6% hydrogen peroxide solution was freshly prepared immediately before use by mixing 45 mL TBS with 5 mL 6% H_2_O_2_ and handled in a fume hood. AEC substrate solution was prepared by mixing 5 mL distilled water with Reagent 1 (72 μL), Reagent 2 (90 μL), and Reagent 3 (80 μL) according to the manufacturer's instructions and applied in the dark (Vector Laboratories, SK‐4200). All steps were performed at room temperature unless otherwise specified.

### 2.3 Masson's Trichrome Stain

Destained tissue sections from 48 experimental wounds from 24 pigs (Table 1) were stained with MT [20].

### 2.4 Digitization and Defining Regions of Interest

All tissue slides (MT and IHC) were scanned at 20× using an Axioscan 7 scanner with a 20×/0.8 objective (Zeiss, Germany) and imported into QuPath v0.6.0 [21]. The image type was set as H‐DAB and Brightfield‐other for the IHC and MT‐stained tissue slides, respectively. Color deconvolution was performed on the IHC‐labeled tissue slides.

**FIGURE 1:**
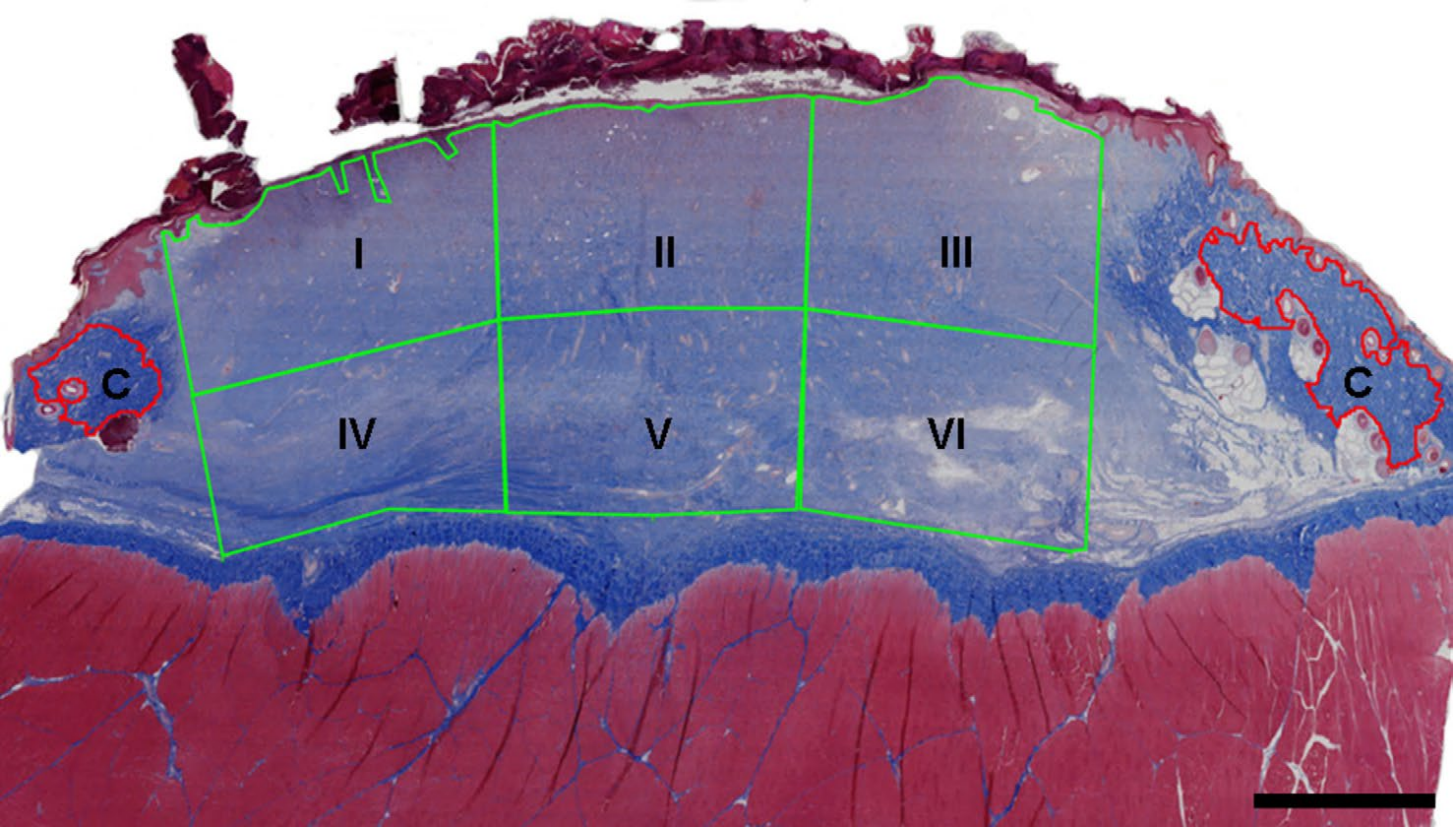
Section of a 10‐day‐old experimental wound stained with Masson's trichrome stain. The granulation tissue within the wound bed was divided into six areas (I–VI). Moreover, two annotations (C) were drawn encompassing the dermal collagen excluding adnexa (control areas) on each side of the wound bed. Bar = 4 mm.

In each of the wounds, six annotations (wound areas I to VI) were drawn as described by Bækgård et al. [5]. In summary, the granulation tissue in the wound was divided into two halves: upper and lower. Thereafter, each half was split into three approximately equal‐sized areas, and annotations were drawn for each of the six wound areas (Figure 1). Moreover, on the MT‐stained tissue slides two annotations encompassing the dermal collagen excluding adnexa, that is, hair follicles and glands, were drawn as control regions on each side of the wound bed (Figure 1).

### 2.5 Quantification of Ki67‐Positive Cells

Detection of Ki67‐positive and ‐negative cells was carried out in QuPath by application of the cell detection command, followed by a random tree object classifier for classification of positive and negative cells. Cell detection with default settings was run on all wounds (*n* = 20) in each of the six wound areas (Figure 1). Then, a subset of the tissue sections (7 wounds from 7 pigs) was randomly selected using the RAND Between function in Excel (Microsoft 365, Microsoft Corporation, Washington, USA) and used for training the object classifier in QuPath. In total, 131 Ki67‐positive and 125 Ki67‐negative cells were annotated and used to train a random trees object classifier using the default settings. The object classifier was run on all experimental wounds (*n* = 20) in each of the six wound areas. Thereafter, the numbers of positive and negative cells in each of the areas of interest were imported into Excel.

To validate the method, one experimental wound from each age group (*n* = 7 wounds) was selected, and all true and false positives and true and false negatives were manually counted in a 300 × 300 μm area in a random wound area selected using the RAND Between function in Excel. Tissue sections used for training were not used for validation.

### 2.6 Quantification of Collagen

**FIGURE 2:**
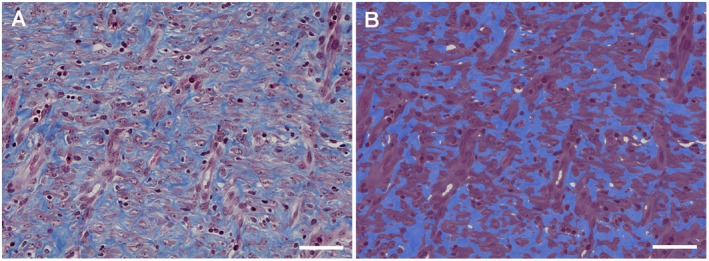
(A) Granulation tissue from a 10‐day‐old experimental wound stained with Masson's trichrome stain. Collagen is stained blue, and cells are stained red. Bar = 140 μm. (B) Same picture as in A after application of an artificial neural network pixel classifier trained to detect collagen (blue) and the cellular components (purple). Bar = 140 μm.

A subset of the MT‐stained tissue sections (26 wounds from 13 pigs) was randomly selected using the RAND Between function in Excel and used for training a pixel classifier in QuPath for collagen segmentation. Two classes were created and named “Collagen” and “Other tissue”. Blue and red pixels were annotated using the polyline tool and set as “Collagen” and “Other tissue”, respectively. In addition, annotations were made in white areas and set as “Ignore”. An artificial neural network pixel classifier was trained at high resolution (0.69 μm/px) using default settings. Annotations were added until the classifier performed acceptable collagen segmentation as verified by visual inspection of each tissue section (Figure 2A,B). The classifier was trained on a total of 1075 annotations (Collagen: 514 annotations, total length 4.9 mm; Other tissue: 561 annotations, total length 4.8 mm).

The pixel classifier was run on all wounds (*n* = 48) in each of the six wound areas and in the control areas.

### 2.7 Statistical Analysis

Statistical analysis was carried out in Excel (Microsoft 365, Microsoft Corporation, Washington, USA) and GraphPad Prism version 10.6.1 (GraphPad Software, Boston, Massachusetts USA, www.graphpad.com). Descriptive statistics were performed to summarize the datasets, and data were checked for normality using QQ plots. A significance level of α = 0.05 was used for all statistical tests.

Ki67‐positive cells: The percentage of Ki67‐positive cells was calculated for each wound area. The upper wound areas (I, II, and III) and the lower wound areas (IV, V, and VI) were combined, respectively, before further analysis. A nested one‐way analysis of variance (ANOVA) was conducted to evaluate differences among the seven age groups in both the upper and lower halves of the wound. In addition, the sensitivity, specificity, and balanced accuracy of the cell detection algorithm were calculated.

Collagen quantification: For each of the areas of interest (wound areas I to VI and the control area, respectively), the fraction of collagen, that is, the area of blue‐stained collagen divided by total area, was imported into Excel. Then the fraction of collagen in wound tissue was divided by the fraction of collagen in the control area (relative collagen fraction) to adjust for any small variations in tissue thickness and stain intensity. For each time point, the mean relative collagen fraction and standard deviations (SD) were calculated. A nested one‐way ANOVA was conducted to evaluate differences among the seven experimental age groups. Tukey's multiple comparisons test was applied to identify specific group differences.

Moreover, to assess whether the ratio between the collagen fraction in the lower and upper part of the experimental wound (collagen ratio) is useful for wound age estimation, the following calculations were carried out. The collagen fractions in areas IV, V, and VI (lower wound areas) were divided by the collagen fraction in areas I, II, and III (upper wound areas), respectively. A nested one‐way ANOVA and Tukey's multiple comparisons test were applied to identify specific differences between the age groups. Moreover, the mean collagen ratio and SD were calculated for each time point (5–35 days).

## 3 Results

### 3.1 Quantification of Ki67‐Positive Cells in Experimental Wounds

A total of 18 wounds from 18 pigs were included. Two wounds (day 5 and day 30) were discarded due to extensive artifacts. A total of 101 wound areas (I–VI) were included for analysis, and seven wound areas were discarded due to artifacts.

The object classifier for detection of Ki67‐positive and ‐negative cells was found to have a sensitivity of 81.6% (CI: 77.8%–85.0%), a specificity of 98.6% (CI: 98.1%–98.9%), and a balanced accuracy of 90.1%. The validation was based on a total of 3749 cells categorized as true positives (*n* = 386 cells), false positives (*n* = 47 cells), true negatives (*n* = 3229 cells), and false negatives (*n* = 87 cells).

**FIGURE 3:**
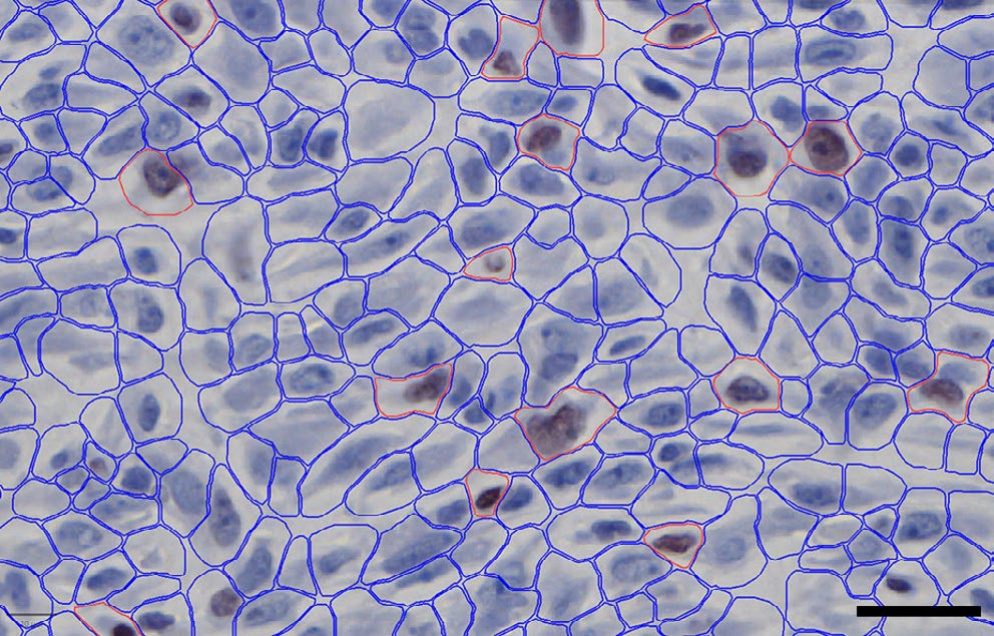
Immunohistochemical detection of cells expressing Ki67 in a 10‐day‐old experimental wound. Positive cells showed brown nuclear staining. Cells were detected using the cell detection command in Qupath and were determined as Ki67‐positive (red outline) or negative (blue outline) by applying a random trees object classifier. Bar = 40 μm.

**FIGURE 4:**
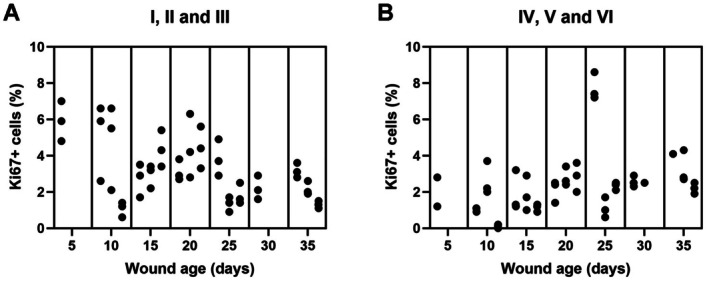
Percentages of positive cells in experimental wounds immunohistochemically stained for Ki67. Each dot represents a wound area (I–VI), and the wound age ranged from 5 to 35 days. No statistically significant difference was found between the age groups (*p* > 0.05). (A) Ki67‐positive cells in the upper half of the wound tissue (wound areas I–III). (B) Ki67‐positive cells in the lower half of the wound tissue (wound areas IV–VI).

No time‐dependent pattern and no significant difference were found between the age groups (Figures 3 and 4A,B).

### 3.2 Quantification of Collagen in Experimental Wounds

A total of 47 wounds from *n* = 24 pigs were included. One wound (day 20) was discarded due to artifacts. A total of 279 wound areas (I–VI) were included for analysis, and three wound areas were discarded due to artifacts.

**FIGURE 5:**
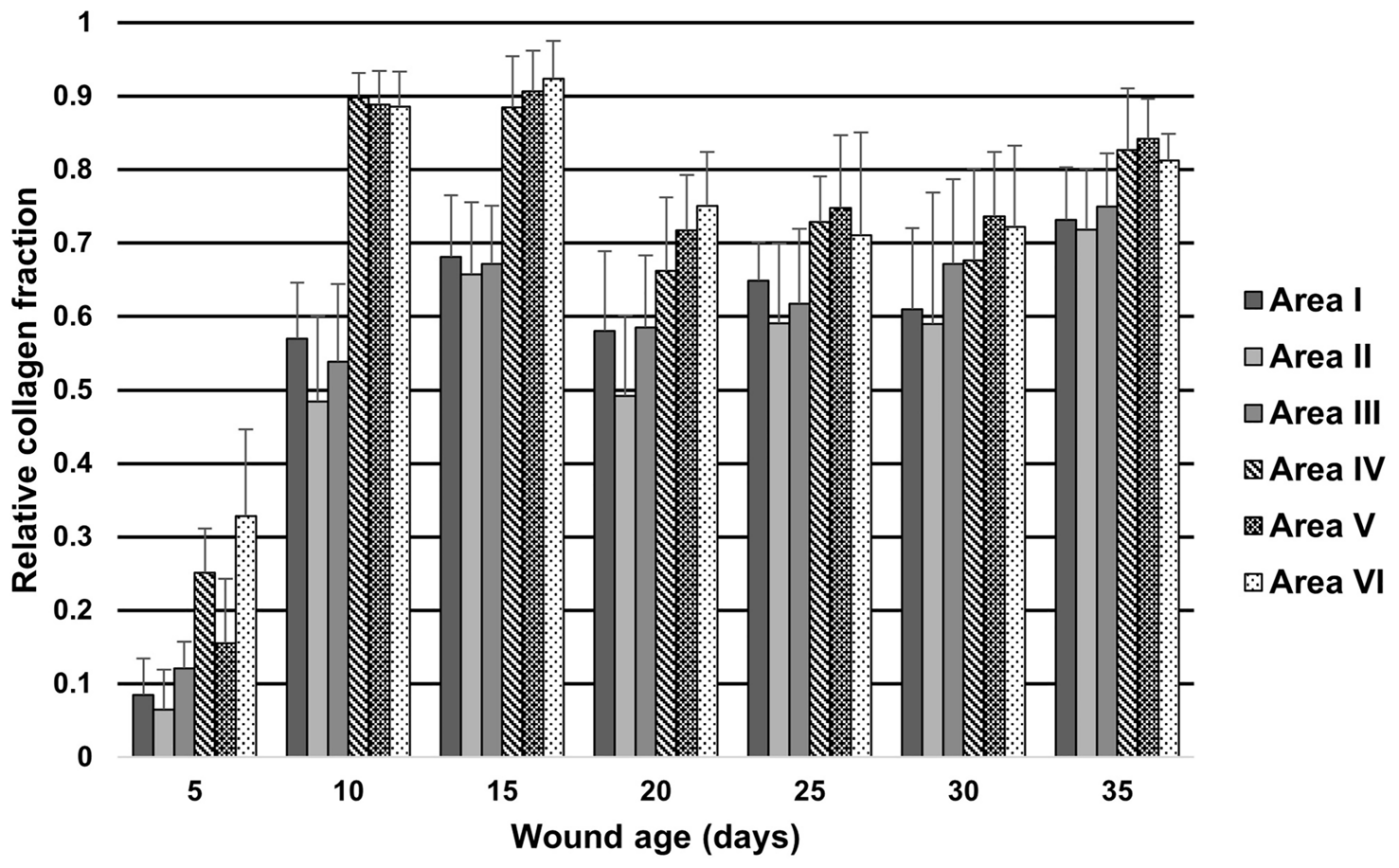
Relative collagen fraction (mean and standard deviation) in areas I–VI in experimental wounds of 5 to 35 days old. The relative collagen fraction was defined as the fraction of collagen in wound tissue divided by the fraction of collagen in the control area. A statistically significant difference was found between the age groups (*p* < 0.05).

The mean fraction of collagen in the control area was 0.92 with a SD of 0.02. The fraction of collagen in the wound tissue relative to the fraction of collagen in the control area (relative collagen fraction) is presented in Figure 5 for each of the wound areas. Significant differences were found between the seven age groups (*p* < 0.0001) (Table 2).

**TABLE 2:** Comparisons of the relative collagen fraction between experimental wounds 5 to 35 days old. A nested one‐way ANOVA showed a significant difference between the experimental age groups (*p* < 0.05). Tukey's multiple comparisons test was applied to identify specific group differences, and *p*‐values lower than 0.05 are listed. Significant differences were found between 5‐day‐old wounds and all other age groups.

Wound age^a^	I	II	II	IV	V	VI
5 vs. 10–35	< 0.0001	< 0.0001	< 0.0001	< 0.0001	< 0.0001	≤ 0.0006
10 vs. 20	—	—	—	0.0034	0.0391	—
10 vs. 25	—	—	—	0.045	—	—
10 vs. 30	—	—	—	0.0227	—	—
10 vs. 35	0.0412	0.0243	0.0125	—	—	—
15 vs. 20	—	—	—	—	0.0125	—
15 vs. 25	—	—	—		0.0499	0.0164
15 vs. 30	—	—	—	0.0346	—	—
20 vs. 35	—	0.0377	—	—	—	—

*Note:* (—) *p*‐values above 0.05.

^a^ Wound age in days.

**FIGURE 6:**
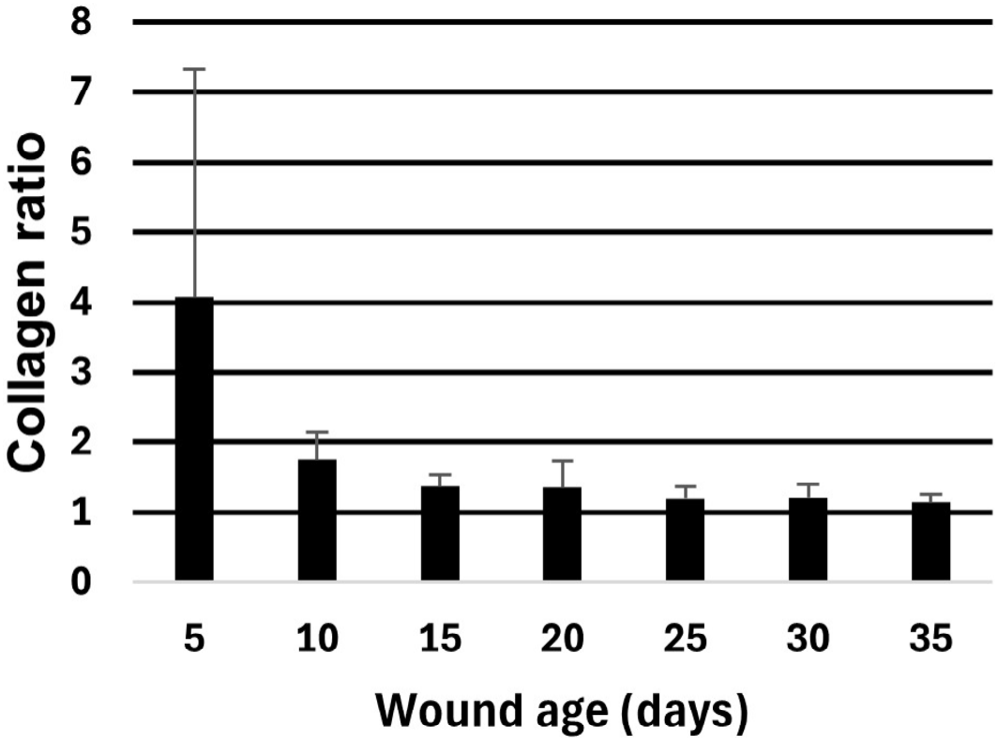
Collagen ratio (mean and standard deviation) between lower and upper parts of the experimental wound tissue, 5 to 35 days old. The collagen ratio was defined as the fraction of collagen in the lower part of the wound tissue (wound areas IV–VI) divided by the fraction of collagen in the upper part of the wound tissue (wound areas I–III).

The fraction of collagen in the lower wound areas was, on average, 1.69 times larger compared to the collagen fraction in the upper part of the wound area (Figure 6). Significant differences were found between 5‐day‐old wounds and the other age groups (*p* < 0.003).

## 4 Discussion

Age assessment of wounds is challenging in both human and veterinary forensic pathology [4, 22]. In forensic porcine cases, the majority of the wounds are chronic, that is, granulation tissue or fibrous scar tissue is present [23]. Veterinary pathologists are asked to assess wound age, which, in a judicial proceeding, may be used to assess the degree of neglect, that is, how long the animal suffered from the wound [2, 4]. Therefore, it is of utmost importance that such age estimates are accurate, precise, and objective.

In the present study, the amount of collagen in porcine wounds generally increased with increasing wound age, as significant differences were seen between day 5 wounds and all other age groups. Moreover, the relative collagen fraction significantly increased on day 35 compared to day 10, but only in the upper wound areas. Timing of collagen formation has previously been described in wound healing studies [2, 8, 17, 24]. In a forensic porcine experimental wound model, the amount of collagen in wounds stained with MT was also found to increase by time [8]. However, in that study, the assessment was descriptive, and collagen was not objectively quantified. In experimental wounds in guinea pigs, collagen fibers were observed from day 3 and increased to the end of the study period of 13 days [24]. Collagen formation was assessed semiquantitatively using light and electron microscopy; however, the method was not described in detail [24]. In canine wounds, collagen has been reported to develop as thin, loosely arranged fibers in 5 to 8‐day‐old wounds and as solid fibers arranged in thick bundles in wounds of 15 to 30 days of age [2]. In that study, the assessment was based on HE, Van Gieson, IHC, and polarized light [2].

In the present study, the relative collagen fraction peaked on days 10 and 15 in the lower wound areas, and significant differences were seen between days 15 and 20 and days 15 and 25, respectively. This pattern has not been described in previous wound healing studies in pigs, dogs, and guinea pigs [2, 8, 24]. However, in sutured experimental wounds in rats, collagen deposition was reported to peak on day six [17]. In that study, collagen was labeled with hydroxyproline, and after extraction, the radioactivity of hydroxyproline was used as a measure of collagen amount [17].

The fraction of collagen in the lower wound areas was, on average, 1.69 times higher compared to the collagen fraction in the upper part of the wound area. This is not surprising, as wounds healing by second intention heal from the wound bed and upwards [5]. However, the collagen ratio between the lower and upper wound areas could only be used to differentiate the 5‐day‐old wounds from older wounds.

In porcine forensic cases, wounds are typically estimated to be from hours, days, several weeks, or months old [23]. Therefore, objective quantification of collagen to assess whether a wound is 5 days old or more is of value for the forensic wound age assessment. However, when differentiating wounds that are, for example, 15 and 35 days old, quantification of collagen amount cannot be used as a standalone method. In a recent study, computational pathology was applied in evaluating the percentages of CD34, CD45, and CD105‐expressing cells in experimental wounds [5]. Both CD45 and CD105 showed significant time‐dependent patterns that could be used as supportive tools for wound age assessment [5]. Therefore, an approach combining several objectively measured time‐dependent markers seems to be promising for improving wound age assessments. Moreover, in future studies, computational quantification of collagen amount should be validated on non‐experimental wounds of known age.

Assessment of collagen in wounds over time has been based mostly on traditional light microscopy and semiquantitative evaluation [2, 8, 24]. However, histological evaluation is to some extent subjective and dependent on the experience of the pathologist [5, 6, 7]. The pixel classifier trained and applied in the present study enables objective measurement of collagen formation in wounds, which is of value in forensic investigations, where age estimations should be as objective as possible.

Tissue thickness and staining intensity must be considered when quantifying the collagen amount. In the present study, the dermis excluding hair follicles and glands was chosen as the reference area, as it was assumed to be unaffected by the wound. This assumption was supported by the fact that the collagen fraction showed little variation in the dermis between tissue sections and between pigs.

In all wounds, < 9% of the cells were Ki67‐positive. The sensitivity, specificity, and balanced accuracy of the object classifier were acceptably high. However, due to the very high number of negative cells relative to positive cells, the specificity of the classifier was correspondingly high.

The proliferation activity, assessed by the percentage of Ki67‐positive cells, did not differ significantly between the age groups. However, there appeared to be a trend of decreasing proliferative activity with increasing wound age in the upper wound areas. The number of observations varied between the age groups, and the limited sample size may explain the lack of statistical significance. However, there is a high variation within each age group, which may render quantification of proliferative activity unsuitable for age assessment of porcine wounds. This is in accordance with a study of canine wounds in which Ki67‐positive cells could not be associated with wound age [2]. However, in rats with experimental dry‐ice burn wounds, mitotic activity of the epidermis showed a time‐dependent pattern with a peak on day 4 [15].

## 5 Conclusions

Application of an artificial neural network pixel classifier trained to differentiate between collagen and cellular components is an objective method for forensic wound age assessment. However, to obtain a higher precision, the method should be used as a supportive tool in combination with other time‐dependent markers. The relative collagen fraction in experimental wounds displayed a significant time‐dependent pattern, where the amount of collagen increased as the wound age increased. Moreover, the collagen ratio between the lower and upper wound areas was able to differentiate the 5‐day‐old wounds from older wounds. Although the proliferation activity was quantified acceptably by the algorithm, proliferation activity assessed by the percentage of Ki67‐positive cells was not suitable for age assessment of porcine wounds.

## Funding

The study was funded by the Independent Research Fund Denmark, Grant ID: https://doi.org/10.46540/2067‐00003B.

## Ethics Statement

The study and the procedures were approved by the Danish Animal Inspectorate. 2023‐15‐0201‐01363.

## Conflicts of Interest

The authors declare no conflicts of interest.

## Data Availability

The data are available from the corresponding author on reasonable request.
